# Supplementary material for: Closed-loop control of a GaAs-based singlet-triplet spin qubit with 99.5% gate fidelity and low leakage
Source: Nat Commun. 2020 Aug 18;11:4144. doi: 10.1038/s41467-020-17865-3 (PMC7434764; doi:10.1038/s41467-020-17865-3)
Supplement: Supplementary file 1 — Supplementary Information [file 41467_2020_17865_MOESM1_ESM.pdf]

Supplementary Information

Closed-loop control of a GaAs-based singlet-triplet spin qubit with 99.5 % gate fidelity and low leakage

Cerfontaine *et al.* (2020)

SUPPLEMENTARY FIGURES

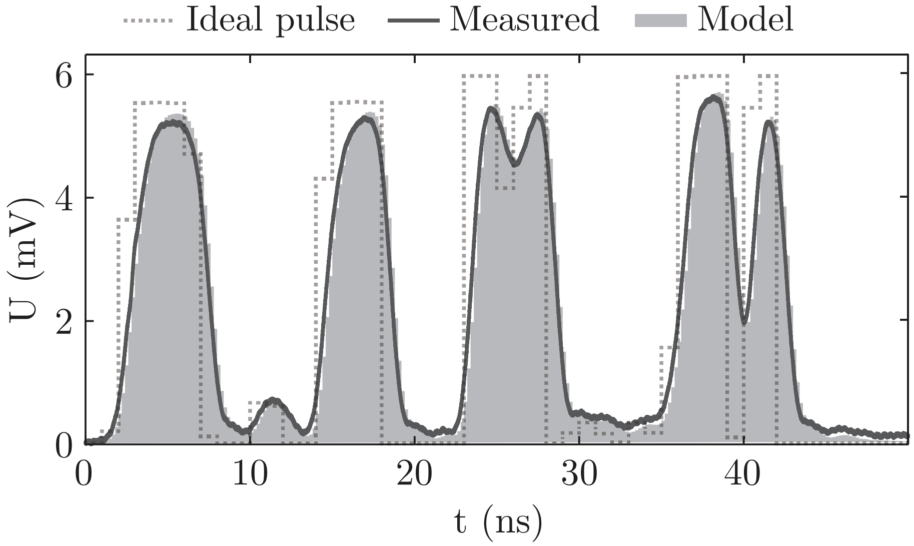

Supplementary Figure 1: Pulse model. A filter based on the step response of our system is applied to nominally piece-wise constant pulses (dashed line) to predict the actual signal arriving at the PCB where the GaAs sample is mounted. Except for long-time transients (not shown) the actual signal (solid line) is accurately reproduced by the model (shaded area).

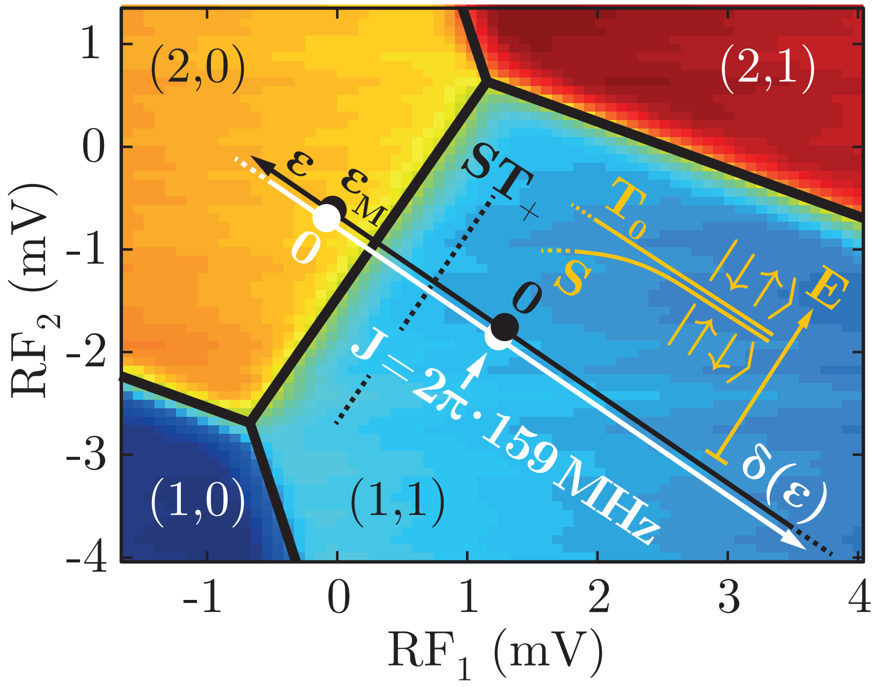

Supplementary Figure 2: Charge stability diagram. Different charge occupations are indicated as a function of both RF gate voltages by (n, m) with n (m) electrons in the left (right) dot. $\delta = \epsilon_\mathrm{M} - \epsilon$ (white) denotes the distance between the measurement point at ϵ_M and the point given by the detuning ϵ (black). $\epsilon = 0$ is defined as the point where $J(\epsilon) = 2\pi \cdot 159\,\mathrm{MHz}$. For large ϵ (low δ) the exchange interaction is turned on, as indicated in the orange energy diagram. The approximate position of the S-T_+ transition is indicated by a dashed line.

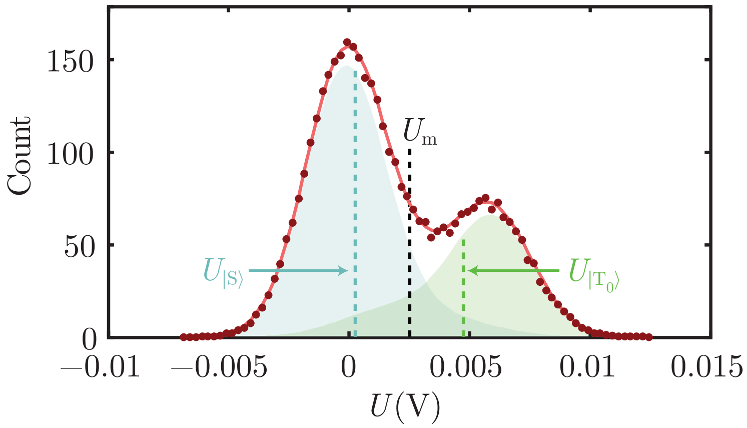

Supplementary Figure 3: Readout calibration. We fit a readout model (light red)[1] to the binned readout voltages of an entire buffer (dark red). The fitted underlying distributions of singlet and triplet voltages are shown in blue and green, respectively. The mean of each distribution is indicated by a dashed line. Extending previous work[1], relaxation (from triplet to singlet) and excitation (from singlet to triplet) are taken into account. The voltages corresponding to singlet $U_{|\mathrm{S}\rangle}$, triplet $U_{|\mathrm{T_0}\rangle}$ and the completely mixed state U_{M} are indicated.

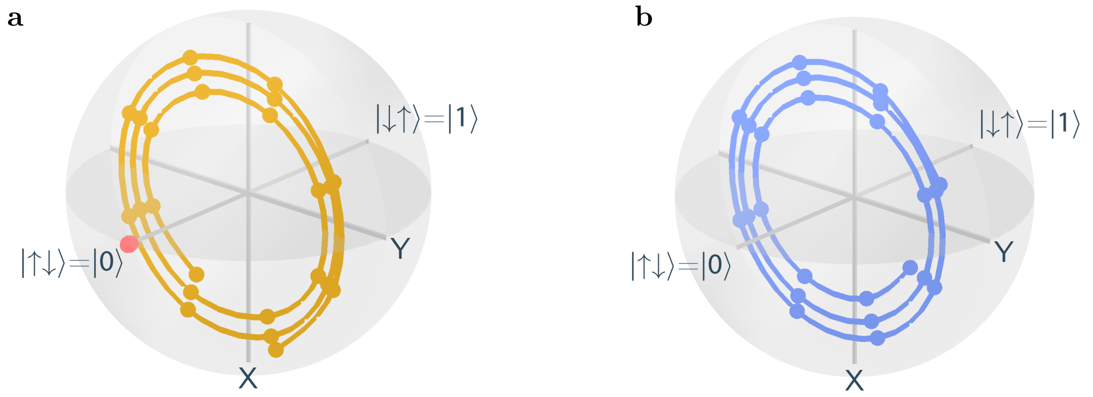

Supplementary Figure 4: Self-consistent state tomography. FID experiments at $J = 0$. The states indicated by dots are reconstructed from an overcomplete set of information, using six different measurements. **a** The state $|0\rangle$ shown by the red dots is an eigenstate and does not evolve. This is different for the initial state of the other FID experiment and the state shown in blue in panel **b**.

SUPPLEMENTARY TABLES

Supplementary Table 1: Theoretical infidelity contributions. The different noise contributions to $\varphi = 1 - \mathcal{F}$ are obtained using Monte Carlo simulations (1000 time traces)[2] for the gates used in Figure 3 . All charge noise below 1 MHz is included in $\varphi_{\epsilon,\text{slow}}$, using noise strengths from Supplementary Note 3. Faster charge noise is taken into account in $\varphi_{\epsilon,\text{fast}}$. The infidelity due to hyperfine noise, $\varphi_{\Delta B_z}$, is much smaller than the charge noise contributions. The first two columns show results for the original numerically-optimized gates which perfectly realize the target gates in the absence of noise. The other two columns contain the results for the experimentally tuned gates, calculated using the same theoretical model as the first two columns. Due to systematic discrepancies between the theoretical qubit model and the actual experiment, the calculated unitary infidelity φ_U is rather high. Due to the GSC tuning, φ_U does not directly affect the final gates but can be used as a measure of how much tuning is required to obtain them from the initial guess.

	Optimized numerically		Tuned experimentally	
	$\pi/2_x$	$\pi/2_y$	$\pi/2_x$	$\pi/2_y$
$\varphi_{\epsilon,\text{fast}}$	2.9×10^{-4}	2.3×10^{-4}	1.7×10^{-4}	2.1×10^{-4}
$\varphi_{\epsilon,\text{slow}}$	1.1×10^{-3}	5.5×10^{-3}	1.6×10^{-3}	3.1×10^{-3}
$\varphi_{\Delta B_z}$	1.2×10^{-4}	8.4×10^{-5}	7.6×10^{-3}	2.2×10^{-3}
φ_U	2.6×10^{-12}	6.8×10^{-12}	1.5×10^{-1}	5.1×10^{-1}
φ	1.4×10^{-3}	6.2×10^{-3}	9.0×10^{-3}	5.3×10^{-3}

Supplementary Table 2: Clifford operations. Sequences of primitive gates used to perform Clifford operations. $\bar{x}, \bar{y}$ and $\bar{z}$ denote the negative x-, y- and z-axis, respectively.

Primitive gates	Clifford gates
$(\pi/2_y)^4$	$(\mathbb{1})$
$(\pi/2_x)^2$	(π_x)
$(\pi/2_y)^2$	(π_y)
$(\pi/2_x)^2 \circ (\pi/2_y)^2$	(π_z)
$(\pi/2_y) \circ (\pi/2_x)$	$(\pi/2_y) \circ (\pi/2_x)$
$(\pi/2_y)^3 \circ (\pi/2_x)$	$(\pi/2_{\bar{y}}) \circ (\pi/2_x)$
$(\pi/2_y) \circ (\pi/2_x)^3$	$(\pi/2_y) \circ (\pi/2_{\bar{x}})$
$(\pi/2_y)^3 \circ (\pi/2_x)^3$	$(\pi/2_{\bar{y}}) \circ (\pi/2_{\bar{x}})$
$(\pi/2_x) \circ (\pi/2_y)$	$(\pi/2_x) \circ (\pi/2_y)$
$(\pi/2_x)^3 \circ (\pi/2_y)$	$(\pi/2_{\bar{x}}) \circ (\pi/2_y)$
$(\pi/2_x) \circ (\pi/2_y)^3$	$(\pi/2_{\bar{y}}) \circ (\pi/2_x)$
$(\pi/2_x)^3 \circ (\pi/2_y)^3$	$(\pi/2_{\bar{x}}) \circ (\pi/2_{\bar{y}})$
$(\pi/2_x)$	$(\pi/2_x)$
$(\pi/2_x)^3$	$(\pi/2_{\bar{x}})$
$(\pi/2_y)$	$(\pi/2_y)$
$(\pi/2_y)^3$	$(\pi/2_{\bar{y}})$
$(\pi/2_x)^3 \circ (\pi/2_y)^3 \circ (\pi/2_x)$	$(\pi/2_z)$
$(\pi/2_x) \circ (\pi/2_y)^3 \circ (\pi/2_x)^3$	$(\pi/2_{\bar{z}})$
$(\pi/2_y) \circ (\pi/2_x)^2$	$(\pi/2_y) \circ (\pi_x)$
$(\pi/2_y)^3 \circ (\pi/2_x)^2$	$(\pi/2_{\bar{y}}) \circ (\pi_x)$
$(\pi/2_x) \circ (\pi/2_y)^2$	$(\pi/2_x) \circ (\pi_y)$
$(\pi/2_x)^3 \circ (\pi/2_y)^2$	$(\pi/2_{\bar{x}}) \circ (\pi_y)$
$(\pi/2_x) \circ (\pi/2_y) \circ (\pi/2_x)$	$(\pi/2_x) \circ (\pi/2_y) \circ (\pi/2_x)$
$(\pi/2_x) \circ (\pi/2_y)^3 \circ (\pi/2_x)$	$(\pi/2_x) \circ (\pi/2_{\bar{y}}) \circ (\pi/2_x)$

Supplementary Table 3: Randomized benchmarking fits. Fit coefficients (with statistical errors) for a self-consistent fit of Supplementary Equations (16-17) to our randomized benchmarking experiments.

$l/3.75$	0.0013 ± 0.0003
$r/3.75$	0.0050 ± 0.0004
A	0.5840 ± 0.0269
B	0.2747 ± 0.0227
χ^2/dof	1.2

SUPPLEMENTARY NOTES

Supplementary Note 1

Experimental setup. This work was performed using two different samples. The first sample was used to establish the calibration routine and measure the Bloch sphere trajectories (see Supplementary Note 17 and Figure 2 d-e). The gate fidelities were obtained in a second sample with lower charge noise. For this reason, this supplementary information focuses on the second sample. Details about the first sample can be found in Ref. 3.

For both samples, the gate structure and electrical setup are the same as in Ref. 4. In the second sample, the S-T_0 qubit is defined in a GaAs/$Al_{0.33}Ga_{0.67}As$ heterostructure with a 2DEG depth of 90 nm. For the heterostructure, Si-modulation doping in a 50 nm $Al_{0.33}Ga_{0.67}As$ layer is used. This layer is separated from the 2DEG by a 35 nm $Al_{0.33}Ga_{0.67}As$ spacer layer. The 5 nm GaAs capping layer is also modulation-doped but due to surface pinning no unwanted channel is formed underneath. The gate layout (Figure 2 b) is the same as in Ref. 5 with two dedicated RF gates for high-frequency qubit operations like initialization, readout and gate operations while DC gates are used for static tuning of the qubits.

In our electrical setup, we use separate DC and RF control gates to avoid pulse distortions from bias tees, resulting in a nearly flat frequency response of the RF gates from DC to beyond 100 MHz. The qubit is defined and tuned by applying static voltages on the order of 1 V to the DC gates, while we use RF pulses from a Tektronix AWG5014C arbitrary waveform generator (AWG) on the order of 1 mV (after attenuation) for qubit manipulation. The RF gates are DC-coupled to the AWG with 43 dB attenuation.

Supplementary Note 2

Exchange pulses. For system characterization we measure the AWG response through coaxial cables and attenuators. We use the AWG to apply a long, nominally rectangular pulse to the RF lines, and measure the signal that arrives just before the printed circuit

board (PCB), where the sample is mounted. From the step response we obtain a filter describing the behavior of our system. Applying this filter to arbitrary piece-wise constant pulses reproduces the actual signal very accurately as shown in Supplementary Figure 1 . The measured step response includes the effect of attenuators and coaxial cables. Since the infidelities of the uncalibrated gates are quite large (see discussion in Supplementary Note 6), the signal distortions due to the PCB and the sample itself could be quite sizable but can be addressed by our self-consistent tuning approach.

Parameters for the experimentally motivated model $J(\epsilon) = J_0 \exp(\epsilon/\epsilon_0)$ from Ref. 6 are fitted from the oscillation frequencies of free induction decay (FID) experiments at different ϵ as in Ref. 6, where ϵ describes the voltage change on each RF gate. We fix $J_0 = 2\pi \cdot 159\,\mathrm{MHz}$ so that $\epsilon = 0$ is always defined as the point where $J(\epsilon) = 2\pi \cdot 159\,\mathrm{MHz}$. This is convenient for describing gate operations as these depend primarily on the magnitude of J, but the exact gate voltages where J has a certain value can vary with dot tuning.

To describe this variation, we define a second frame of reference $\delta = \epsilon_\mathrm{M} - \epsilon$. Here, ϵ_M is the position of the measurement point in the $(2,0)$ charge configuration as shown in Supplementary Figure 2 and corresponds to $\delta = 0$. Even if the distance of the measurement point from the $(2,0) - (1,1)$ charge transition remains the same, typically around $0.25\,\mathrm{mV}$, the exact value of ϵ_M depends on dot tuning since $\epsilon = 0$ is defined as the point where $J(\epsilon) = 2\pi \cdot 159\,\mathrm{MHz}$. This leads to shifts of the ϵ coordinate system when the point where $J = 2\pi \cdot 159\,\mathrm{MHz}$ moves. The gate fidelity of 99.50 % presented in the main text was obtained with gates optimized for $\epsilon_0 = 0.60\,\mathrm{mV}$ and $\epsilon_\mathrm{M} = 1.67\,\mathrm{mV}$.

Supplementary Note 3

Noise. In addition to the characterization of $J(\epsilon)$ and the step response of our setup, we extract approximate hyperfine and charge noise levels from free induction decay (FID) and spin echo (SE) experiments.

For hyperfine noise we find $T_2^* = 80\,\mathrm{ns}$, with ΔB_z stabilized at a setpoint of approximately $2\pi \cdot 42.1\,\mathrm{MHz}$ by dynamic nuclear polarization[7]. This value is slightly smaller than $T_2^* =$

94 ns reported in Ref. 7. Note that coherence times of about 0.9 ms can be achieved by sophisticated dynamical decoupling sequences[8], indicating a large contribution from low-frequency noise. Instead of measuring T_2^*, we can also measure the fluctuations in ΔB_z directly. For a variety of dot tunings, which affect the effectiveness of the dynamic nuclear polarization pulses[7], we find standard deviations between $\sigma_{\Delta B_z} = 2\pi \cdot 2.2\,\mathrm{MHz}$ and $\sigma_{\Delta B_z} = 2\pi \cdot 3.4\,\mathrm{MHz}$ (after removing data where ΔB_z stabilization did not work properly). The gate fidelity of 99.50 % presented in the main text was obtained with $\sigma_{\Delta B_z} = 2\pi \cdot 2.8\,\mathrm{MHz}$, which corresponds to $T_2^* = 80\,\mathrm{ns}$. Since ΔB_z fluctuates on a much longer timescale[9] than gate operations, we always treat hyperfine noise as quasistatic throughout this work.

For charge noise, we perform FID and SE experiments at various detunings. At $J(\epsilon) = 2\pi \cdot 119\,\mathrm{MHz}$ we obtain $T_2^* = 77\,\mathrm{ns}$ and $T_2^{\mathrm{echo}} = 6.1\,\mu\mathrm{s}$. For these FID and SE experiments we also determined $\epsilon_0 = 0.69\,\mathrm{mV}$ and $\epsilon_{\mathrm{M}} = 1.59\,\mathrm{mV}$ so that we can approximately calculate the charge noise sensitivity in our device at $J(\epsilon) = 2\pi{\cdot}119\,\mathrm{MHz}$ to $dJ/d\epsilon = 2\pi{\cdot}160\,\mathrm{MHz/mV}$.

We use the relation

$$T_2^* = \frac{\sqrt{2}}{\sigma_\epsilon dJ/d\epsilon} \tag{1}$$

given in Ref. 10 to calculate our sample's charge noise standard deviation to $\sigma_\epsilon = 1.8 \times 10^{-5}\,\mathrm{V}$. To deduce the amplitude S_ϵ^0 of the high-frequency noise spectrum from T_2^{echo}, we assume that the noise spectrum can be described by $S_\epsilon(f) = S_\epsilon^0/f^\beta$ with $\beta = 0.7$ as measured in Ref. 6 for frequencies $50\,\mathrm{kHz} \leq f \leq 1\,\mathrm{MHz}$. Due to the proportionality

$$\frac{1}{S_\epsilon^0} \propto \left(T_2^{\mathrm{echo}}\right)^{1+\beta} \left(\frac{dJ}{d\epsilon}\right)^2 \tag{2}$$

following from Ref. 10, we can estimate the amplitude of the high-frequency charge noise spectrum by relating our measurements of T_2^{echo} and $dJ/d\epsilon$ to those from Ref. 6, which were given as $T_2^{\mathrm{echo}} \approx 7.5\,\mu\mathrm{s}$, $dJ/d\epsilon \approx 2\pi \cdot 150\,\mathrm{MHz/mV}$ and $\sqrt{S_\epsilon^0} = 2.8 \times 10^{-8}\,\mathrm{V}/\sqrt{\mathrm{Hz}}$. For our sample, we then find $\sqrt{S_\epsilon^0} = 3.2 \times 10^{-8}\,\mathrm{V}/\sqrt{\mathrm{Hz}}$.

These noise estimates are used in Supplementary Note 6 to calculate the gate fidelities we expect to reach theoretically. For this analysis, we extrapolate the spectrum as white since the functional form of the noise spectrum is not known above 1 MHz. Even for this worst-case estimate, high-frequency charge noise does not appear to be limiting gate fidelities, as discussed in Supplementary Note 6. Fast charge noise would only become relevant if the

noise spectral density S_ϵ^0 was about 4 times larger. Thus, a more detailed analysis of the high-frequency charge noise spectrum was not conducted for this work.

Even though the charge noise in our device is larger than in Ref. 6, we observe a better quality factor of 726 compared to 585 for a comparable charge noise sensitivity $dJ/d\epsilon \approx 2\pi \cdot 160\,\mathrm{MHz/mV}$, likely due to a higher curvature ϵ_0.

Supplementary Note 4

Bloch sphere convention. Throughout this work, we use the convention that $J(\epsilon)$ coincides with the Bloch sphere's y-axis and ΔB_z with the z-axis.

If $J(\epsilon)$ were chosen to point along the x-axis of the Bloch sphere, our $\pi/2_x$ ($\pi/2_y$) gate would actually be a $\pi/2$ rotation around the negative y-axis (positive x-axis).

Supplementary Note 5

Numerical pulse optimization. The pulse optimization we perform for this work is similar to Ref. 2. For completeness, we now provide a short summary and highlight any differences.

First, we characterize the step response of our electrical setup and measure J_0, ϵ_0 and ΔB_z as described in Supplementary Note 2. We also determine the coherence of our qubit as described in Supplementary Note 3. Consequently, we use this information as a model for the numerical pulse optimization of piece-wise constant detuning pulses with N_seg segments. Each pulse segment is 1 ns long, corresponding to the sampling rate of our AWG. In the following, ϵ_j with $j = 1 \ldots N_\mathrm{seg}$ denotes the detuning in the jth segment. The actual control pulse arriving at the qubit is determined by applying a filter to the piece-wise constant pulse ϵ_j (see Supplementary Note 2). This is even more realistic than the procedure outlined in Ref. 2 where a purely exponential model was used to account for finite rise times.

We also choose slightly different bounds than Ref. 2 and constrain the detuning to $1.00\,\mathrm{mV} \leq$

$\delta \leq 4.00\,\mathrm{mV}$. The upper bound of δ corresponds to the $(1,1)$ charge regime with separated electrons. At this point $J \ll \Delta B_z$ is essentially turned off and ϵ is at the baseline ϵ_{min} mentioned in the main text. The lower bound was chosen to avoid the anticrossing of the singlet state with the $|\mathrm{T}_+\rangle$ state during gate operation. This anticrossing occurs at large detunings ϵ, typically at a distance $\delta \approx 0.65\,\mathrm{mV}$ from the measurement point M. The crossing is also indicated in Figure 1 and Supplementary Figure 2 . We avoid pulsing near or across this anticrossing as it would lead to a significant portion of the singlet state being converted to the leakage state $|\mathrm{T}_+\rangle$. We found that keeping a distance of around $0.35\,\mathrm{mV}$ leads to low gate leakage rates while retaining sufficient dynamic range in J. For future experiments, it would be helpful to further examine the process driving leakage so that it can be captured and minimized directly in the numerical pulse optimization.

Furthermore, at least the last 4 ns of each gate were set to ϵ_{min} (which corresponds to $\delta = 4.00\,\mathrm{mV}$) to ensure that the pulse has decayed to ϵ_{min} before another gate is applied. If the decay is not perfect, transients from the previous gate will affect the next gate. Since such gate bleedthrough[11] causes additional errors which depend on the sequence of gates and not just the gates themselves, it is harder to detect and correct than purely gate-dependent errors. Thus, it is best to minimize gate transients whenever possible.

For the pulse optimization, explicitly time-dependent Hamiltonians $H(J(\epsilon(t)), \Delta B_z) = \frac{\hbar J(\epsilon(t))}{2}\sigma_x + \frac{\hbar \Delta B_z}{2}\sigma_z$ are approximated as piecewise constant on 0.2 ns intervals. This discretization greatly simplifies the calculation of $U(t, t_0) = \mathcal{T}\exp\left(-\frac{i}{\hbar}\int_{t_0}^{t} H(t')\,dt'\right)$ and incurs only small systematic errors on the order of 10^{-3} (in fidelity). These can be calibrated in experiments by using a self-consistent gate set characterization and calibration protocol (which we will from now on abbreviate as GSC). Using this approximation, we can also calculate the effect of quasistatic noise in ϵ and ΔB_z. All we have to do is to repeat the calculation several times using $H(J(\epsilon(t) + d\epsilon), \Delta B_z + d\Delta B_z)$, where $d\epsilon$ ($d\Delta B_z$) is sampled discretely from a Gaussian distribution with standard deviation σ_ϵ ($\sigma_{\Delta B_z}$). It is a little bit more involved to include fast noise with an arbitrary noise spectral density in a computationally efficient manner. We choose to use a first-order perturbative approach based on filter functions[12] which is fast enough for numerical optimization.

We then combine these methods with the Levenberg-Marquardt algorithm (LMA) to search

for $\pi/2_x$ and $\pi/2_y$ pulses with maximal fidelity $\mathcal{F}$, taking systematic errors and the major decoherence sources into account. Further details regarding the exact implementation of the optimization are given in Ref. 2. An example of the resulting pulses $\epsilon_j^g, j = 1 \ldots N_{\text{seg}}$ for two gates $g = \pi/2_x$ and $g = \pi/2_y$ can be found in Figure 2 a.

Supplementary Note 6

Fidelity estimation. Using the noise model described in Supplementary Note 3, we use Monte Carlo (MC) simulations as in Ref. 2 to determine the average gate fidelity of the gate set used in Figure 3 . For hyperfine noise we use $\sigma_{\Delta B_z} = 2\pi \cdot 2.8\,\text{MHz}$.

We list the theoretical noise contributions to the infidelity $\varphi = 1 - \mathcal{F}$ in Supplementary Table 1. In the first two columns we use the original numerically optimized pulses and find an average gate fidelity of $(99.62 \pm 0.01)\,\%$, where the error indicates the uncertainty from MC. In the other columns we also calculate the fidelity of the experimentally tuned pulses to $(99.29 \pm 0.02)\,\%$. The large systematic errors of up to $50\,\%$ indicate inaccuracies in our control model, including the setup's impulse response and nonlinear transfer function $J(\epsilon)$. For all columns in Supplementary Table 1 the infidelity is dominated by quasistatic charge or quasistatic hyperfine noise. While the numerically optimized pulses are insensitive to hyperfine noise, this robustness is not maintained for the experimentally tuned pulses. We attribute this to the large systematic deviations caused by the erroneous control model, which destroy the gates' dynamical decoupling characteristics, at least for this theoretical calculation.

It is reasonable to assume that applying the numerically optimized pulses to our experiment also incurs systematic errors on the order of $30\,\%$. Indeed, after performing scaling of the pulses, randomized benchmarking (RB) yields average primitive gate fidelities of around $10\,\%$. Before scaling of the pulses, RB cannot really be measured, which is consistent with errors on the order of tens of percent. Removing most of these errors with GSC then yields the experimentally obtained fidelity of $(99.50 \pm 0.04)\,\%$. Even though this figure still contains residual systematic errors, it is in good agreement with our theory predictions, which do not include systematic deviations. Since the experimentally observed fidelity would

be higher without systematic errors, this indicates that our noise model can be used to obtain good estimates on the achievable fidelities.

To assess the potential for further improvement, it is also instructive to consider the effect of stretching or compressing one of our numerically optimized pulses in time. Doing so results in distinct scaling laws of the contributions to φ from different noise types. For fast charge noise, the scaling law also depends on the spectral noise density $S_\epsilon(f) \propto 1/f^\beta$. When we change the total duration T of a pulse and accordingly adjust $J(\epsilon)$ and ΔB_z so that the same unitary is still realized, we find

$$\varphi_{\epsilon,\text{fast}} \propto T^{\beta-1}, \tag{3}$$

$$\varphi_{\epsilon,\text{slow}} \sim \text{const}, \tag{4}$$

$$\varphi_{\Delta B_z} \propto T^2, \tag{5}$$

assuming $\varphi \ll 1$. $\varphi_{\Delta B_z}$ increases with T since ΔB_z decreases with T while the standard deviation of ΔB_z remains the same. Thus, the error of the phase acquired due to ΔB_z increases with T. For slow charge noise we additionally need to consider $dJ/d\epsilon$ which decreases with J. Consequently, the standard deviation of $J(\epsilon)$ decreases with T and $\varphi_{\epsilon,\text{slow}}$ is approximately constant. For high-frequency charge noise the form of the noise spectrum given by the exponent β needs to be taken into account since the frequency range most relevant for the gate changes with $1/T$. Using the filter function formalism[12], $\varphi_{\epsilon,\text{fast}} \propto T^{\beta-1}$ can be derived.

From Supplementary Equations (3-5) we calculate which duration T is optimal given that hyperfine noise contributes 2.0×10^{-3} to the overall infidelity, and assuming a 2.0×10^{-4} contribution from high-frequency charge noise according to Supplementary Table 1. For $\beta = 0.7$, making the gates 6.2 times faster would lead to approximate hyperfine and fast charge noise contributions of 3.5×10^{-4} and 5.2×10^{-5}, improving the average gate fidelity by 1.8×10^{-3}. For $\beta = 1$ the hyperfine noise contribution can be suppressed without increasing the contribution from fast charge noise. Thus, the fidelity could be easily improved to 99.8 % in both cases.

Supplementary Note 7

Readout and initialization pulses. The FID and SE experiments used for measuring hyperfine noise (see Supplementary Note 3) are performed with singlet initialization and readout.

For all other experiments, we prepare the state $|\uparrow\downarrow\rangle$ adiabatically. To this end, we first decrease ϵ quickly and diabatically jump over the S-T$_+$ transition to avoid mixing with the $|\mathrm{T}_+\rangle$ level. Then, we slowly ramp ϵ down to ϵ_{min}. In this way, $J(\epsilon)$ is turned off adiabatically since $J(\epsilon_{\mathrm{min}}) \ll \Delta B_z$. For readout, we slowly sweep ϵ from ϵ_{min} up to a point before the S-T$_+$ transition. Then, we jump directly to the measurement point ϵ_{M}. We cross the S-T$_+$ transition diabatically in order to avoid mixing with the $|\mathrm{T}_+\rangle$ level. Since $J(\epsilon)$ is turned back on adiabatically we measure in the basis $(|\uparrow\downarrow\rangle, |\downarrow\uparrow\rangle)$.

Supplementary Note 8

Data acquisition. Data is acquired in buffers with a duration of approximately 100 ms. Each buffer contains ~ 20 repetitions of $N \sim 1000$ pulse sequences, and we typically measure between 10 to 1000 buffers in order to ensure good averaging over the slowly decorrelating ΔB_z. Every pulse sequence consists of an adiabatic initialization pulse to prepare $|\uparrow\downarrow\rangle$, qubit manipulation pulses and adiabatic readout, which maps singlet and triplet probabilities to $|\uparrow\downarrow\rangle$ and $|\downarrow\uparrow\rangle$ probabilities. In the following we refer to singlet probabilities $p(|0\rangle)$ whenever we mean the probability of measuring $|\uparrow\downarrow\rangle$.

After each buffer, polarization pulses[7] are applied to stabilize the hyperfine gradient ΔB_z. Once in about 10^3 measurements we also read out a completely mixed state and a triplet state as additional references. The mixed state is prepared by rotating a $|\uparrow\downarrow\rangle$ state around five axes with different $J(\epsilon)$ for times on the order of a µs, much longer than the coherence time T_2^* of the qubit. For perfectly orthogonal axes and complete dephasing, this procedure would prepare a completely mixed state. The triplet state is prepared by precession of a singlet state in the stabilized hyperfine field ΔB_z.

We discriminate between singlet and triplet states by Pauli spin blockade. Using spin to charge conversion[13], the resistance of an adjacent sensing dot depends on the spin state and can be determined by RF-reflectometry[14]. In this manner, we obtain different readout voltages for singlet and triplet states, $U_{|\mathrm{S}\rangle}$ and $U_{|\mathrm{T}_0\rangle}$, but cannot distinguish between $|\mathrm{T}_0\rangle$ and the triplet states $|\mathrm{T}_\pm\rangle$.

We only switch on the RF readout power during measurements while leaving it off during manipulation. The timing of this switching is chosen carefully to minimize the influence of transients on the measured voltages.

The voltages measured in a buffer, which contains about 20 repetitions of a sequence of $N \sim 1000$ pulses, are processed in two ways:

1. All measured voltages are binned, irrespective of the pulse sequence or repetition they are associated with, to obtain histograms of the readout values.
2. Measured voltages corresponding to repetitions of the same pulse sequence are averaged, yielding N averaged voltages U.

For optimal ΔB_z stabilization, we use a different number of pulses N for different experiments. Fewer pulses lead to faster repetition rates of the DNP pulses and thus to better ΔB_z stabilization. Thus, we used $N = 1008$ for the calibration and $N = 640$ for RB.

Since data requirements differ between the various experiments, details about further data processing and our readout calibration are given in separate notes, specifically in Supplementary Note 10 and Supplementary Note 12.

Supplementary Note 9

Data postselection. During RB, we measure ΔB_z by performing an FID experiment alongside each repetition. We use this information to discard data where dynamic nuclear polarization (DNP) was not stable and deviated by more than $\pm 2\pi \cdot 15\,\mathrm{MHz}$ from the setpoint of ΔB_z at about $2\pi \cdot 42.1\,\mathrm{MHz}$. This approximately corresponds to a 5σ interval around the

setpoint and does not change the measured gate fidelities. While it typically takes about ten measurements to automatically reach the setpoint of 42.1 MHz, it is usually not necessary to discard any data afterwards.

For the RB data presented in the main text, only the first 15 data points were removed. Afterwards, ΔB_z did not deviate by more than 15 MHz from the target so that no further post-selection occured.

Supplementary Note 10

Readout calibration. For self-consistent state tomography (see Supplementary Note 17 and Figure 2 d-e), we convert measured voltages to singlet probabilities $p(|0\rangle)$ by fitting the bimodal histogram[1] obtained from binning the measurement data (see Supplementary Note 8).

In contrast to Ref. 1, we observe excitation from $|\mathrm{S}\rangle$ to $|\mathrm{T_0}\rangle$ as well as relaxation from $|\mathrm{T_0}\rangle$ to $|\mathrm{S}\rangle$ in the first sample. Since the standard model[1] does not fit our data well, we extend it by including an excitation rate e from $|\mathrm{S}\rangle$ to $|\mathrm{T_0}\rangle$ in addition to the relaxation rate r. In separate experiments we measure T_1 decays in the $(2,0)$ charge region where readout is performed. From this data, we determine T_1 and the steady state voltage V_{SS} to which states decay after long times in the $(2,0)$ charge region. T_1 and V_{SS} are then used as additional fixed parameters in the fit of the histograms. The resulting fits typically look like the one in Supplementary Figure 3 . $U_{|\mathrm{S}\rangle}$ and $U_{|\mathrm{T_0}\rangle}$ can then be calculated from the fit parameters and correspond to singlet probabilities of $p(|0\rangle) = 1$ and $p(|0\rangle) = 0$.

We can now convert the averaged voltages U to singlet probabilities $p(|0\rangle) \in [0,1]$ using a linear transformation $p(|0\rangle) = \frac{1}{2}\langle\sigma_z\rangle + \frac{1}{2} = \frac{c}{2}(U+s) + \frac{1}{2}$ with coefficients c and s obtained from the fit. Inaccuracies from fitting of the single shot histogram should not affect the tomography results as self-consistent state tomography is robust to state preparation and measurement (SPAM) errors.

For our benchmarking experiments (see Supplementary Note 18 and Figure 3) performed in the second sample, we use a similar calibration. In contrast to the first sample, we observe

much less excitation from $|S\rangle$ to $|T_0\rangle$. Hence, we can use the standard model without excitation[1] to fit the single shot histograms. In addition, long gate sequences create a significant $|T_+\rangle$ population. For the readout calibration we have so far neglected that other states than $|T_0\rangle$ and $|S\rangle$ might be populated. We have attempted to include $|T_+\rangle$ explicitly in the fit model but found that this introduces too many additional parameters. While we have not quantitatively analyzed the error due to this issue, we suspect that the suboptimal contrast in Figure 3 might be related.

As RB and all other benchmarking experiments performed in this work are insensitive to SPAM errors, our readout calibration does not need to be especially accurate or precise. Specifically, the suboptimal contrast does not affect any figures of merit reported in this work.

Supplementary Note 11

State preparation and measurement errors. In this note, we argue that state preparation errors can be mapped to measurement errors. Hence, we only need to consider the latter in following discussions.

By definition, state preparation does not suffer from systematic errors as it defines the computational basis state $|0\rangle$. However, the purity of the initial state can be finite due to stochastic errors from incomplete relaxation to the ground state[15] or from a combination of imperfect adiabaticity and dephasing. As a check, we prepare $|\uparrow\downarrow\rangle$, let it evolve for varying times (between 0 ns and 24 ns in 1 ns increments) and read out along different axes than z using diabatic pulses before the adiabatic readout. Since the resulting readout voltages do not show sinusoidal oscillations and correspond to a high contrast of approximately 97 % (obtained by fitting histograms as explained in Supplementary Note 10), we conclude that imperfect adiabaticity is not a problem for our initialization.

Since we use the same sweep speed for the adiabatic ramps for state initialization and readout, our readout should be along the same axis as the state preparation. We check whether this assumption is justified by preparing different initial states than $|\uparrow\downarrow\rangle$ (using

diabatic pulses after the adiabatic initialization) and letting them evolve in the hyperfine field at $J(\epsilon_{\mathrm{min}})$ for varying times (between 0 ns and 24 ns in 1 ns increments) before adiabatic readout. Again, the resulting readout voltages do not show sinusoidal oscillations. Thus, we can deduce that readout and initialization axes coincide well with the rotation axis at $J(\epsilon_{\mathrm{min}})$ since in neither case oscillations could be observed. Thus, readout and initialization are performed along the same axis (up to measurement noise, which could have been larger than a small sinusoidal signal). Note that readout and initialization can still suffer from different stochastic errors.

Since it is impossible to discriminate stochastic state preparation errors from readout errors, the state preparation can be treated as perfect and all state preparation errors can be mapped to our adiabatic measurement[15]. Adiabatic state preparation and adiabatic measurement are performed along the same axis as explained above.

Supplementary Note 12

Gate Set Calibration (GSC). For optimization with GSC, the averaged voltages U_i corresponding to the error syndromes S_i (as defined in Table 1) do not need to be explicitly converted to singlet probabilities $p(|0\rangle)$ or $\langle\sigma_z\rangle$. Instead, the norm of $\tilde{U_i} = U_i - U_{\mathrm{M/T}}$ can be optimized without further calibration. In this expression $U_{\mathrm{M/T}}$ corresponds to U_{M} if $i = 1 \ldots 6$, otherwise the voltage U_{T} of the reference $|\mathrm{T}_0\rangle$ state is used. The reference triplet state $|\mathrm{T}_0\rangle$ is subject to decoherence because it is prepared by letting $|\mathrm{S}\rangle$ evolve in the hyperfine field at $J(\epsilon_{\mathrm{min}}) \ll \Delta B_z$. U_{T} is obtained by correcting the actual measurement result U'_{T} for the approximate loss in contrast of the triplet preparation using the transformation $U_{\mathrm{T}} = U'_{\mathrm{T}} + b(U'_{\mathrm{T}} - U_{\mathrm{M}})$ with $b \sim 10^{-2}$ chosen according to T_2^* measurements. The exact choice of b is not crucially important as long as reductions in the decoherence of the gate set result in a reduction of S_7 and S_8. Furthermore, S_7 and S_8 should be of comparable magnitude as the other syndromes for optimal convergence. This can be achieved by choosing appropriate weights w_7 and w_8 in Supplementary Equation (15).

It is helpful to compensate fluctuations of the measurement contrast with the aid of histograms to speed up convergence. With c and s defined as in Supplementary Note 10 this

yields

$$\tilde{S}_i = c\tilde{U}_i \tag{6}$$
$$= c(U_i + s) - c(U_{\mathrm{M/T}} + s) \tag{7}$$
$$= S_i - S_{\mathrm{M/T}}, \tag{8}$$

which is the same relation as given in the main text.

Supplementary Note 13

Error syndromes. The gate sequences from Table 1 used to extract the error syndromes are modified versions of the previously published bootstrap tomography[16]. GSC uses the information obtained from the error syndromes in a specific manner explained in Supplementary Note 15. As described in the methods section of the paper and in Supplementary Note 7, we adiabatically prepare $|\uparrow\downarrow\rangle$ before applying any of the gate sequences from Table 1. At the end of each gate sequence we perform an adiabatic readout, allowing us to discriminate between $|\uparrow\downarrow\rangle$ and $|\downarrow\uparrow\rangle$.

Possible errors include decoherence and systematic errors, which can be categorized as over-rotation and off-axis errors. We use the same parameterization for systematic errors as in Refs. 2 and 16 and denote the rotation-angle error of the $\pi/2_x$ ($\pi/2_y$) gate by 2ϕ (2χ) while axis-errors are described by n_y, n_z (v_x, v_z). Using this notation, the unitary operator of the erroneous $\pi/2_x$ gate can be written as

$$U_x = \exp\left[-i(\boldsymbol{n}\cdot\boldsymbol{\sigma})(\pi/2 + 2\phi)/2\right] \tag{9}$$
$$\text{with } \boldsymbol{n} = \left(\sqrt{1 - n_y^2 - n_z^2},\ n_y,\ n_z\right)^{\top}. \tag{10}$$

Likewise, the unitary operator of the erroneous $\pi/2_y$ gate is given by

$$U_y = \exp\left[-i(\boldsymbol{v}\cdot\boldsymbol{\sigma})(\pi/2 + 2\chi)/2\right] \tag{11}$$
$$\text{with } \boldsymbol{v} = \left(v_x,\ \sqrt{1 - v_x^2 - v_z^2},\ v_z\right)^{\top}. \tag{12}$$

The first six sequences are sufficient to extract all systematic errors, and we introduce two additional sequences to explicitly probe for decoherence.

Supplementary Note 14

Robustness to measurement errors. In this note we discuss GSC's robustness to SPAM errors. As pointed out in Supplementary Note 11, all adiabatic state preparation errors can be mapped to the adiabatic measurement. Thus, we can focus solely on measurement errors in this note. Furthermore, we have discussed in Supplementary Note 11 that the state preparation axis and measurement axis align in our system. Since GSC works with only one readout axis and does not require precalibrated gates, the relevant readout errors can be captured by a reduction in contrast δ_c ($0 \leq \delta_c \leq 1$) and a shift δ_s ($-1 \leq \delta_s \leq 1$) of the measurement results.

These parameters capture that measuring the error syndrome S_i will generally not result in the correct voltage U_i but instead yield $U_i' = \delta_c(U_i + \delta_s)$. Note that some further constraints on δ_c and δ_s are needed to ensure that the measurement stays physical. As described in Supplementary Note 12, GSC optimizes

$$\tilde{S}_i = c(\delta_c U_i + \delta_c \delta_s + s) - c(\delta_c U_\mathrm{M} + \delta_c \delta_s + s) \tag{13}$$

$$= c\delta_c(U_i - U_\mathrm{M}) \tag{14}$$

so that shifts δ_s are of no consequence.

Changes in the measurement contrast δ_c will lead to slower convergence as discussed in Supplementary Note 16. However, the feedback loop should still converge to the same target as $c\delta_c(U_i - U_\mathrm{M})$ is always zero for a perfect gate set, irrespective of δ_c. As pointed out in Supplementary Note 12, histograms of the measured voltages can be used to determine c so that $c\delta_c$ is approximately constant. Hence, GSC can be made robust to fluctuations δ_c as long as $c\delta_c$ does not change significantly between GSC iterations.

Supplementary Note 15

Feedback loop. Before the feedback loop is started, we perform a scaling analysis to compensate shifts in $J(\epsilon)$. To this end, we independently scale the ϵ pulses of both gates. Specifically, we scale the amplitude minus the baseline, $\epsilon_j^g - \epsilon_{\min}$, by $\pm 10\%$ in increments of $2\,\%$ and measure all 8 error syndromes for each scale factor. We start the iteration with the best scale factor chosen separately for each gate.

For the iterative control loop we use the Levenberg-Marquardt Algorithm (LMA), which requires derivatives of the objective function to compute an update. Throughout this work, the LMA iteratively solves the problem

$$\min_{\epsilon_j^g} \left| \left(\, \tilde{\mathbf{S}}_{\mathbf{1-6}}(\epsilon_j^g), \; w_7 \tilde{S}_7(\epsilon_j^g), \; w_8 \tilde{S}_8(\epsilon_j^g) \, \right) \right|^2, \tag{15}$$

where $\tilde{\mathbf{S}}_{\mathbf{1-6}}$ is a six-component row vector consisting of the first six error syndromes $\tilde{S}_i, i = 1 \ldots 6$ (see Table 1 and Supplementary Note 12). This minimization problem is a slightly modified version of Eq. (2) from Ref. 2. In the beginning of GSC they are chosen as $w_7 = w_8 = 1$ but after a few iterations (after iteration 9 in Figure 2 c) the decoherence signals are completely turned off by setting $w_7 = w_8 = 0$. This procedure is designed to improve convergence of the LMA algorithm[17], motivated by the observation that systematic errors can still be tuned close to a solution while this is more difficult for decoherence-induced errors. We use the LMA because we found in simulations that derivative free methods such as the Nelder-Mead algorithm (NMA) typically take at least an order of magnitude more iterations than the LMA to converge well. This of course only holds true as long as derivatives can be reliably measured. Minimizing the number of iterations is important as pulse updates on our arbitrary waveform generator (AWG) and reference measurements take about 2 min during which we cannot acquire new data. On the other hand we can perform on the order of 10^5 measurements per second once pulses have been uploaded to the AWG. Consequently, we would like to minimize the number of iterations rather than the number of measurements.

We estimate the derivatives required for the LMA from measurement data using forward finite differences with a fixed step size. It is important to choose a finite difference step size

which is large enough so that the numerical derivatives are not dominated by measurement noise. However, the estimation error becomes larger for large step sizes. The optimum step size depends on the curvature and magnitude of the objective function. Since curvature and magnitude depend on the point in parameter space and are not generally known, we simulate GSC, and find a step size $h = 0.2\epsilon_0 \dots 0.3\epsilon_0$ to be optimal for a noise level of 1×10^{-2} of the measured singlet probability $p(|0\rangle)$. These values also work well in our experimental setup.

The time for each iteration varies between 4 min and 2 h since we measure much longer once low error syndromes have been reached in order to improve the accuracy of the finite differences. This also includes the time needed for pulse updates on the AWG and post-processing of the data on a standard desktop computer. The overall time can be further optimized by using more efficient schemes to estimate the Jacobian.

Supplementary Note 16

Convergence. Not considering abrupt changes in dot parameters due to charge rearrangements, the speed of convergence is similar to what we expect from simulations[2], where SPAM errors were completely disregarded. As mentioned in Supplementary Note 14, GSC should be robust to slow shifts of the readout voltages. But slow changes in the measurement contrast δ_c will affect $\tilde{S}_i = c\delta_c(U_i - U_\mathrm{M})$ if not compensated by a different choice of c so that $c\delta_c$ remains approximately constant. If this is the case, derivatives $d\tilde{S}_i/d\epsilon_j^g$ cannot be reliably measured and suboptimal updates of the pulse parameters ϵ_j^g will be performed. Since the speed of convergence is similar to the simulations, choosing c from histogram fits seems to work well.

As in the simulations[2], convergence stops when the noise floor has been reached. For our experiments, we measured enough data points for each different gate sequence so that a noise level of approximately 4×10^{-3} of $\langle\sigma_z\rangle = 2p(|0\rangle) - 1$ is obtained. With this noise level we find that convergence typically stops when the signals $\tilde{S}_i$ are on the order of 5×10^{-2}. However, the simulations converge to lower syndromes $\tilde{S}_i \approx 5 \times 10^{-3}$ for the same noise level[2]. This discrepancy could arise from specific forms of gate-related decoherence which affects $\tilde{S}_i$. Additionally, charge drifts in our sample could lead to changes in the detuning ϵ (which is

equivalent to a change in J_0) and hence slowly shift the target during the optimization.

Infrequent charge rearrangements change $J(\epsilon)$ and affect which pulse parameters ϵ_j^g are optimal. If charge rearrangements occur within an optimization run, we proceed as follows: Initially, we wait for the dot to return to the previous configuration and discard the data recorded in the wrong dot configuration. If the original configuration has not been restored after a short while ($\sim$ hours), we manually tune the dot back to a working configuration. As gate performance is very sensitive to the exact functional form of $J(\epsilon)$, manual tuning of the dot typically leads to a deterioration of gate fidelity. Depending on the amount of tuning required, we can either continue with the optimization (as done after iteration 11 shown in Figure 2 c) or we need to start from the beginning.

Supplementary Note 17

Self-consistent state tomography. Following Ref. 15, we calibrate the self-consistent state tomography using five FID experiments for five different initial states with the exchange interaction switched off ($\epsilon = \epsilon_{\text{min}}$). For each free evolution, multiple points are read out along six different measurement axes. This overcomplete set allows us to determine the five initial states and six measurement operators from a self-consistent fit. Following the argument in Supplementary Note 11, we assume for the fit that the axes of adiabatic state preparation and measurement align, and that all initialization errors can be mapped to the measurement.

Any unknown state is consequently read out using the six measurement operators known from the calibration. Since this again results in overcomplete information as only three linearly independent measurement operators would be needed, the unknown state is determined as the best fit (in the least-squares sense) to the overcomplete information. The states for different evolution times of FID experiments reconstructed in this way are shown in Supplementary Figure 4 , showing good agreement with the expected trajectories.

The gate trajectories in Figure 2 d-e are obtained in the same way. The gate operation is stopped in increments of 1 ns and the resulting final state is read out. There is a small caveat however. The state tomography was calibrated for readout pulses which start at

$\epsilon = \epsilon_{\text{min}}$. While the gate operation returns to ϵ_{min} in the end, intermediate points may be at $\epsilon > \epsilon_{\text{min}}$. Due to finite bandwidth effects, the measurement operator will then vary with ϵ and might be different than in the calibration. Thus, readout is only reliable if the previous operation has already reached ϵ_{min} before the state is read out (including finite rise times). Such concatenation errors contribute to the deviations between the trajectories shown in Figure 2 d-e when the exchange interaction is switched on.

Supplementary Note 18

Randomized benchmarking. For randomized benchmarking (RB), we compose Clifford operations from the primitive gate set according to Supplementary Table 2, resulting in an average of 3.75 primitive gates per Clifford. This procedure is commonly used in other works which use RB for gate characterization, e.g. by Ref. 18.

In addition to standard RB, we also extract loss rates using a modified randomized benchmarking protocol[19] (LRB) which omits the last Clifford gate from each RB sequence, resulting in the data shown in blue in Figure 3 . We choose this protocol over a very similar protocol designed to extract coherent leakage rates since we can not perform operations with the required experimental control relative to leakage levels[20].

Once the calibration procedure has reached sufficiently low error syndromes, we measure both RB and LRB in consecutive experiments, obtaining the data given in Figure 3 . We then self-consistently fit the singlet probabilities

$$p_{\text{RB}}(x) = \frac{A}{2}\left[1 - l(x-1)\right] \cdot \left[1 + (1-2r)^x\right] + B \tag{16}$$

$$p_{\text{LRB}}(x) = \frac{A}{2}\left[1 - l(x-1)\right] + B \tag{17}$$

to the red RB and blue LRB data set, respectively. Since we measure only small populations of the leakage subspace, we neglect the return of leakage states to the computational subspace in these equations. In this approximation, $1 - l(x-1)$ corresponds to the probability of remaining in the computational subspace to linear order in lx. Since the decay rate r refers to the RB decay in the computational subspace, we multiply by $1 - l(x-1)$ in the first

equation. Furthermore, fitting a joint visibility parameter A and offset B ensures that the fit is well-conditioned even for low loss rates.

The fit yields the coefficients shown in Supplementary Table 3 from which the average fidelity $(99.50 \pm 0.04)\,\%$ and loss rate $(0.13 \pm 0.03)\,\%$ for the primitive gate set from Figure 3 are extracted. Since all qubit operations are performed well away from any charge transitions, we can rule out that the electron is lost during gate operations. Thus, we interpret the loss rate as incoherent leakage out of the computational subspace into the leakage subspace. While the origin of leakage is not firmly established, we conjecture that leakage into $|T_+\rangle$ is dominant since our gate pulses approach the S-T_+ transition.

In addition to leakage, another caveat in RB are gate-dependent errors which are likely to be present here since the Clifford operations are generated from a limited primitive gate set. If we add a term for gate-dependent errors of the form $C(x-1)(1-2r)^{x-2}$ in Supplementary Equation (16)[21,22] inside the second set of square brackets, the other fit coefficients change only slightly while C turns out to be insignificantly different from zero. Furthermore, numerical simulations suggest that even in the case of gate-dependent errors or $1/f$-noise, benchmarking provides a "better than a factor-of-2 estimate of the average error rate" (p.11 of Ref. 23). Thus, gate-dependent errors should not significantly influence our fidelity estimate based on RB.

Similar to RB, the derivation of the fit model for the loss protocol assumes gate-independent noise. While we cannot check whether all formal prerequisites of the loss protocol are satisfied, the linear model fits the data well. The joint fit with the RB data ensures that the SPAM coefficients A and B can be determined well, so that the extracted rate itself is reliable. Furthermore, decoherence in the code space is nearly complete for gate counts greater than 150 so that leakage is the only physical process driving the slow decay.

SUPPLEMENTARY REFERENCES

[1] Barthel, C., Reilly, D., Marcus, C., Hanson, M. & Gossard, A. Rapid Single-Shot Measurement of a Singlet-Triplet Qubit. *Physical Review Letters* **103**, 160503 (2009).

[2] Cerfontaine, P., Botzem, T., DiVincenzo, D. P. & Bluhm, H. High-Fidelity Single-Qubit Gates for Two-Electron Spin Qubits in GaAs. *Physical Review Letters* **113**, 150501 (2014).

[3] Cerfontaine, P. *et al.* Feedback-tuned noise-resilient gates for encoded spin qubits. *Preprint at http://arxiv.org/abs/1606.01897* (2016).

[4] Botzem, T. *et al.* Quadrupolar and anisotropy effects on dephasing in two-electron spin qubits in GaAs. *Nature Communications* **7**, 11170 (2016).

[5] Shulman, M. D. *et al.* Demonstration of entanglement of electrostatically coupled singlet-triplet qubits. *Science* **336**, 202–205 (2012).

[6] Dial, O. E. *et al.* Charge noise spectroscopy using coherent exchange oscillations in a singlet-triplet qubit. *Physical Review Letters* **110**, 146804 (2013).

[7] Bluhm, H., Foletti, S., Mahalu, D., Umansky, V. & Yacoby, A. Enhancing the Coherence of a Spin Qubit by Operating it as a Feedback Loop That Controls its Nuclear Spin Bath. *Physical Review Letters* **105**, 216803 (2010).

[8] Malinowski, F. K. *et al.* Notch filtering the nuclear environment of a spin qubit. *Nature Nanotechnology* **12**, 16–20 (2016).

[9] Reilly, D. J. *et al.* Measurement of Temporal Correlations of the Overhauser Field in a Double Quantum Dot. *Physical Review Letters* **101**, 236803 (2008).

[10] Dial, O. E. *et al.* Supplement: Charge noise spectroscopy using coherent exchange oscillations in a singlet-triplet qubit. *Physical Review Letters* **110**, 1–4 (2013).

[11] Kelly, J. *et al.* Optimal Quantum Control Using Randomized Benchmarking. *Physical Review Letters* **112**, 240504 (2014).

[12] Green, T., Uys, H. & Biercuk, M. J. High-Order Noise Filtering in Nontrivial Quantum Logic Gates. *Physical Review Letters* **109**, 020501 (2012).

[13] Petta, J. R. *et al.* Coherent manipulation of coupled electron spins in semiconductor quantum dots. *Science* **309**, 2180–2184 (2005).

[14] Reilly, D. J., Marcus, C. M., Hanson, M. P. & Gossard, A. C. Fast single-charge sensing with

a rf quantum point contact. *Applied Physics Letters* **91**, 89–92 (2007).

[15] Takahashi, M., Bartlett, S. D. & Doherty, A. C. Tomography of a spin qubit in a double quantum dot. *Physical Review A* **88**, 022120 (2013).

[16] Dobrovitski, V. V., de Lange, G., Ristè, D. & Hanson, R. Bootstrap Tomography of the Pulses for Quantum Control. *Physical Review Letters* **105**, 077601 (2010).

[17] Cerfontaine, P., Otten, R. & Bluhm, H. Self-Consistent Calibration of Quantum-Gate Sets. *Physical Review Applied* **13**, 044071 (2020).

[18] Barends, R. *et al.* Superconducting quantum circuits at the surface code threshold for fault tolerance. *Nature* **508**, 500–503 (2014).

[19] Wallman, J. J., Barnhill, M. & Emerson, J. Robust Characterization of Loss Rates. *Physical Review Letters* **115**, 060501 (2015).

[20] Wallman, J. J., Barnhill, M. & Emerson, J. Robust characterization of leakage errors. *New Journal of Physics* **18**, 043021 (2016).

[21] Magesan, E., Gambetta, J. M. & Emerson, J. Characterizing quantum gates via randomized benchmarking. *Physical Review A* **85**, 042311 (2012).

[22] Magesan, E. *et al.* Efficient Measurement of Quantum Gate Error by Interleaved Randomized Benchmarking. *Physical Review Letters* **109**, 080505 (2012).

[23] Epstein, J. M., Cross, A. W., Magesan, E. & Gambetta, J. M. Investigating the limits of randomized benchmarking protocols. *Physical Review A* **89**, 062321 (2014).
