# Supplementary material for: Closed-loop control of a GaAs-based singlet-triplet spin qubit with 99.5% gate fidelity and low leakage
Source: Nat Commun. 2020 Aug 18;11:4144. doi: 10.1038/s41467-020-17865-3 (PMC7434764; doi:10.1038/s41467-020-17865-3)
Supplement: Supplementary file 2 — Description of Additional Supplementary Files [file 41467_2020_17865_MOESM2_ESM.pdf]

Description of Additional Supplementary Files

Supplementary Data 1 :

This archive contains the randomized benchmarking data and the leakage randomized benchmarking data (corresponding to Fig. 3 in the main text) as recorded and automatically post-processed by our measurement framework. The data format is explained in the included README file.
